# The effect of three‐dimensional visualisation on performance in endoscopic sinus surgery: A clinical training study using surgical navigation for movement analysis in a randomised crossover design

**DOI:** 10.1111/coa.13494

**Published:** 2020-01-27

**Authors:** Ellen ten Dam, Herman M. Helder, Bernard F. A. M. van der Laan, Robert A. Feijen, Astrid G. W. Korsten‐Meijer

**Affiliations:** ^1^ Department of Otorhinolaryngology/Head and Neck Surgery University Medical Center Groningen University of Groningen Groningen The Netherlands; ^2^ Institute for Drug Exploration Graduate School of Medical Sciences Groningen University Groningen The Netherlands; ^3^ Cancer Research Center Groningen Graduate School of Medical Sciences Groningen University Groningen The Netherlands

**Keywords:** anterior skull base, endoscopic sinus surgery, imaging, technology, undergraduate education

## Abstract

**Objectives:**

Endoscopic imaging techniques and endoscopic endonasal surgery (EES) expertise have evolved rapidly. Only few studies have assessed the effect of three‐dimensional (3D) endoscopy on endoscopic sinus surgery (ESS). The present study aimed to objectively and subjectively assess the additional value of 3D high‐definition (HD) endoscopy in ESS.

**Design:**

A randomized crossover study of endoscopic surgery performance, using five ESS tasks of varying complexity, performed on Thiel embalmed human specimens.

**Setting:**

Simulated surgical environment.

**Participants:**

Thirty participants, inexperienced in ESS.

**Main outcome measures:**

Performance was assessed using video imaging, surgical navigation and questionnaires. Main outcome measures were as follows: efficiency (defined by time to task completion), distance covered inside the nose, average velocity towards target, accuracy (measured by error rate), and subjective assessment of endoscope characteristics.

**Results:**

During ESS tasks, both efficiency and accuracy did not differ significantly between 2D HD and 3D HD endoscopy. Subjectively, imaging characteristics of the 3D HD endoscope were rated significantly better.

**Conclusions:**

ESS performance of inexperienced participants was not significantly improved by the use of 3D HD endoscopy during ESS tasks, although imaging characteristics of the 3D HD endoscope were rated significantly better. Surgical field characteristics and surgical techniques are likely to influence any additional value of 3D HD endoscopy.

**Keypoints**

- Use of three‐dimensional (3D) high‐definition (HD) endoscopy does not significantly improve surgical performance of inexperienced surgeons compared to two‐dimensional HD during endoscopic sinus surgery tasks, even though 3D HD endoscopy is subjectively rated significantly better.
- When choosing a specific endoscope type, it is recommended to take the specific circumstances in which endoscopic endonasal surgery will be performed into account.
- Surgical navigation can be used to assess surgical performance in endonasal endoscopic (sinus) surgery tasks.

## 1 INTRODUCTION

Over the past decades, endoscopic imaging techniques and endoscopic surgery expertise have improved continuously. Today, a wide range of sinonasal and skull base pathology can be treated successfully with endoscopic endonasal surgery (EES).^1^, ^2^ In EES, two‐dimensional (2D) high‐definition (HD) endoscopes are widely used, and although they provide good quality images of the surgical field, depth perception is limited compared with direct sight or use of a surgical microscope.^3^, ^4^ Specifically during extended EES procedures, when detailed display of anatomical relations is required, the limited depth perception is thought to limit surgical performance.^5^, ^6^

The development of three‐dimensional (3D) endoscopes, that provide surgeons with a stereoscopic view of the surgical field, has proved challenging, especially with optics suitable for endonasal surgery. Early 3D endoscopes had large diameter optics, offered limited image quality and caused side effects to its users.^7^, ^8^ Only in recent years, reliable 3D endoscopes with 4‐5 mm diameter optics became available.^8^, ^9^, ^10^, ^11^, ^12^ After being used predominantly in transnasal neurosurgical procedures, 3D endoscopes have started to find their way into endoscopic sinus surgery (ESS).^5^, ^9^, ^13^, ^14^

Literature specifically addressing the effect of 3D endoscopy on surgical performance in ESS is sparse.^5^, ^9^, ^14^ Most studies assessing the influence of 3D endoscopic imaging on surgical performance focused on laparoscopic surgery or neurosurgical procedures.^15^, ^16^ In laparoscopic surgery, the use of 3D endoscopic imaging reduced the duration of procedures and the amount of error rates.^16^ During neurosurgical procedures in clinical studies, no significant differences in procedure duration or tumour resection were found, while depth perception, spatial orientation, identification of anatomical relations and critical structures subjectively improved with the use of 3D endoscopy.^15^ In a laboratory setting, 3D endoscopy decreased the time to task completion in both laparoscopic and neurosurgery procedures.^11^, ^12^, ^14^, ^17^, ^18^, ^19^, ^20^, ^21^, ^22^ Both expert and novice endoscopic surgeons benefit from 3D visualisation,^15^ although various studies suggest a specific benefit for inexperienced surgeons.^12^, ^14^, ^23^ This can be explained by experienced surgeons already having acquired the ability to effectively translate 2D visual information to 3D perception of the surgical field, thereby reducing the additional value of 3D endoscopy.

It is questionable to what extent the results of neurosurgical and laparoscopy studies of 3D endoscopy are applicable to ESS because surgical performance and the benefits of 2D HD and 3D HD endoscopy are likely to be related to surgical field properties and surgical technique.^6^, ^14^, ^24^ The present study aimed to assess the additional value of 3D HD endoscopy in ESS. A 3D HD and a 2D HD endoscope were used by inexperienced surgeons in a realistically simulated clinical setting. Performance was assessed during various ESS tasks, using both objective and subjective outcomes measures. It was hypothesised that the use of 3D HD endoscopy in ESS would significantly and objectively improve surgical efficiency and accuracy, in addition to the subjective performance.

## 2 METHODS

### 2.1 Ethical considerations

Ethical approval for the performance of the present study was obtained from the Medical Ethical Committee of the University Medical Center Groningen (UMCG). No specific approval was deemed necessary before commencing.

### 2.2 Subjects

This study was performed at the skills centre of the Wenckebach Institute of the UMCG, Groningen, the Netherlands. Medical students of the University of Groningen and residents otorhinolaryngology, neurosurgery and general surgery of the UMCG were invited to participate. Participants were included if they were able to read and write Dutch. Those failing to pass the Titmus circle test (Stereo Optical Company) were excluded. Subjects fulfilling the inclusion criteria were randomised into two groups, either performing endoscopic surgical tasks first with 2D HD and second with 3D HD endoscopy (2D first‐3D second), or in the opposite (3D first‐2D second) order.

### 2.3 Specimen

Two Thiel‐embalmed human cadaver heads were used.^25^, ^26^ On the left side of the nose, an anterior and posterior ethmoidectomy, medial maxillectomy type II and removal of the anterior sphenoid sinus wall were performed.^27^ Titanium screws (Gebrüder Martin GmbH & Co. KG), measuring 1.5 × 6 mm, were placed at the following locations: (a) the entrance of the anterior and posterior ethmoidal artery in the ethmoidal sinus; (b) the entrance of the sphenopalatine artery in the nose; and (c) in the posterior wall of the maxillary and sphenoid sinus. Subsequently, the specimen heads were scanned using a Somatom Force high‐resolution CT scanner and slices of 0.4‐0.6 mm to allow accurate surgical navigation (Siemens Healthcare GmbH).

### 2.4 Technology

#### 2.4.1 Navigation system

A KICK electromagnetic (EM) navigation system (Brainlab AG) was used to track the location, and calculate the distance covered and the velocity of the surgical instrument used. The system contained a standard pointer, and an additional EM sensor that could be attached to the other instruments used. The systems reference sensor was fixed with sutures to the anterior scalp. The location of the pointer or instrument tip within the surgical field was registered seven times per second. The system was installed in accordance with the user manual.

#### 2.4.2 Endoscopic equipment and display

For 3D imaging, a Visionsense VSiii endoscopic system with 4 mm diameter, 0° angled optics (Visionsense, LtD), was used. This endoscope has an interpupillary distance of 1.2 mm and a dynamic focus range of 8‐80 mm. It uses “insect eye” technology—incorporating an array of microlenses integrated into 4 mm optics—to project two separate endoscopic images onto dedicated sections of the camera chip.^9^, ^10^, ^11^, ^28^ The images were displayed on a 24‐inch HD (1920 × 1080 pixels) stereoscopic LED flat screen that offered true stereoscopic images when passive polarising glasses were worn.

For 2D imaging, a Karl Storz 2D HD 0° endoscope (Karl Storz GmbH & Co.) was used. The camera of the endoscope had a maximum resolution of 1920 × 1080 pixels, making it full HD. Images were displayed on a 23‐inch full HD LCD flat screen monitor.

### 2.5 Study design

Five endoscopic surgical tasks (described below) were designed, based on the validated, low‐cost sinus surgery task trainer developed by Steehler et al.^11^, ^12^ Although well designed and easy to use, the simulator provides a simplified model of the surgical environment in which endoscopic surgery is performed. The tasks used in the present study were modified to be performed on a human specimen. To minimise the bias of differences in anatomical knowledge, all participants watched instruction videos showing the actual surgical field and examples of the task execution, before the start of each task.

#### 2.5.1 Task 1) Identification of anatomical landmarks

**Figure 1:**
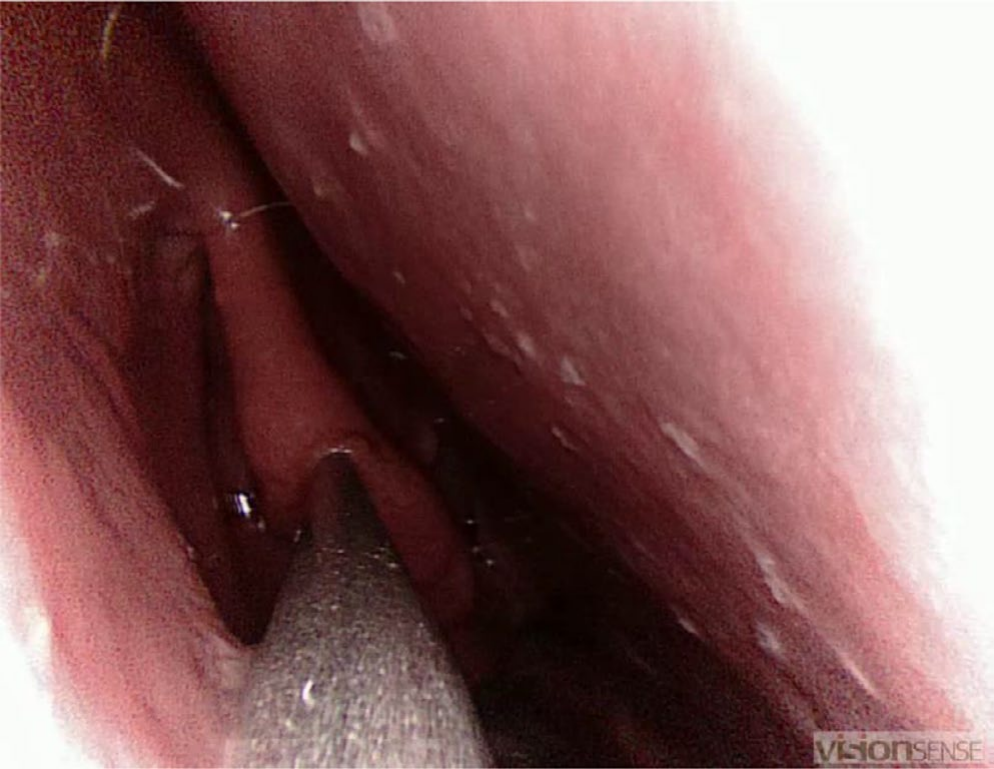
First task: participant placing the navigation system pointer on the middle turbinate (right side of the nose)

Six anatomical landmarks were identified by placing the navigation system probe under direct endoscopic view on the appropriate landmark. Landmarks were identified in the following sequence: head of the middle turbinate, uncinate process, choana upper border, head of the inferior turbinate, ethmoid bulla and most superior point of the semilunar hiatus (Figure 1).

#### 2.5.2 Task 2) Approaching targets

**Figure 2:**
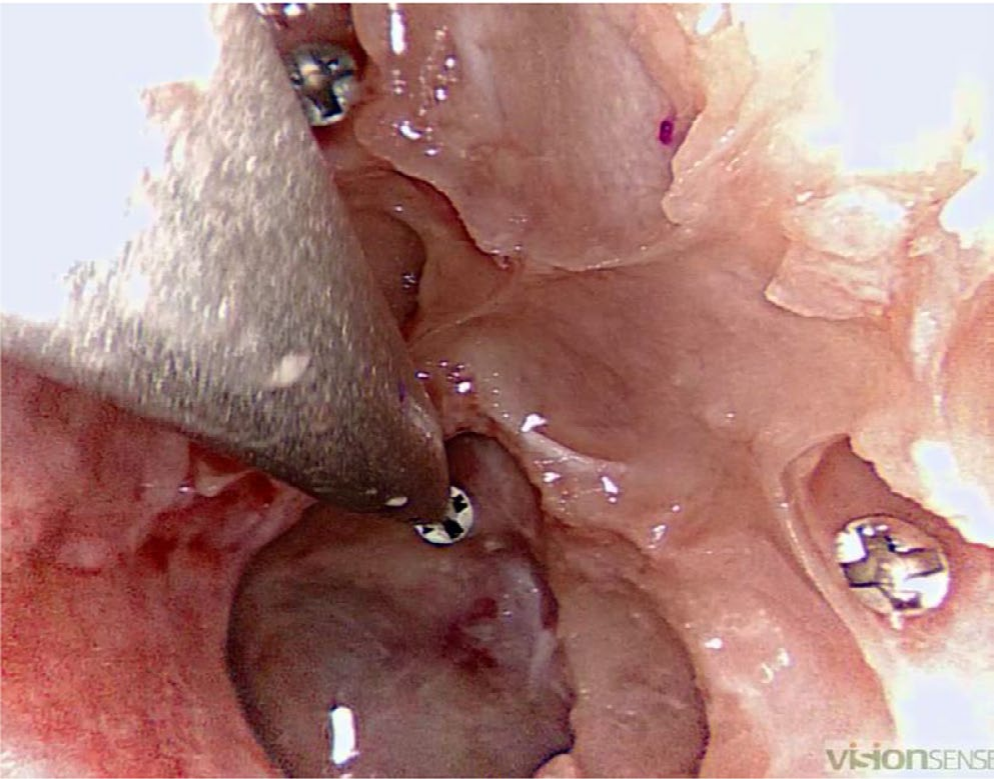
Second task: participant placing the navigation system pointer central in the screw head of a screw placed near the left opticocarotid recess

The navigation system probe was placed exactly in the centre of each of the five screw heads. The following sequence was used: anterior ethmoidal artery, sphenoid sinus, maxillary sinus, posterior ethmoid artery and the nasal entrance of the sphenopalatine artery (Figure 2).

#### 2.5.3 Task 3‐5) Grasping and retrieving objects

**Figure 3:**
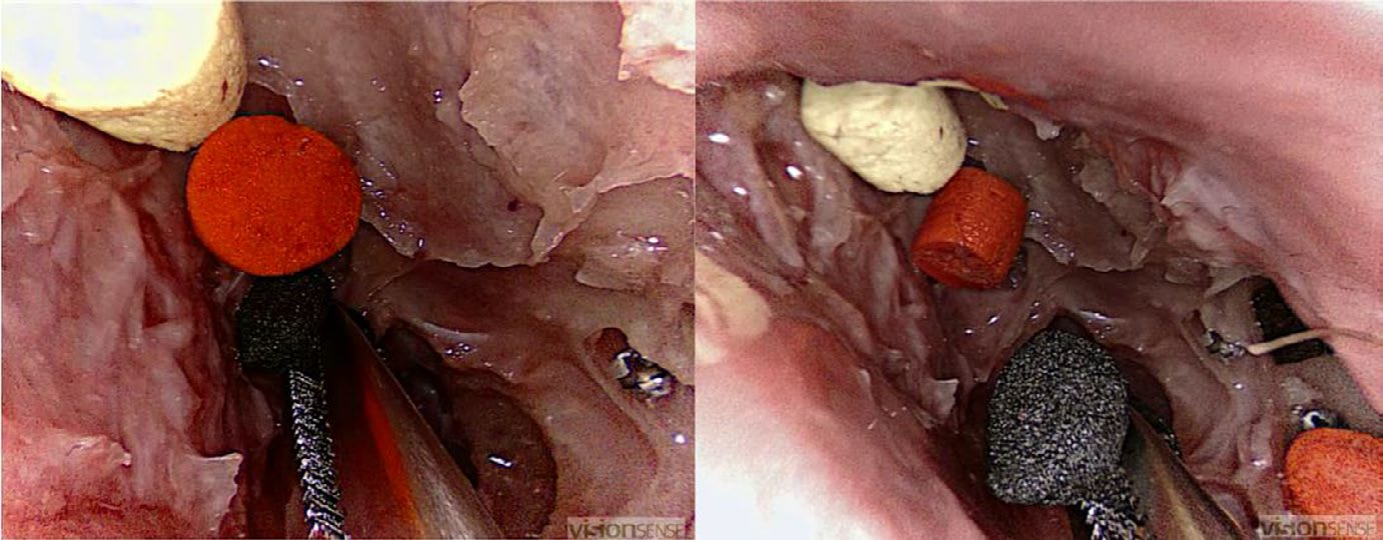
Third task: participant grasping one of the six sponge discs with a straight forceps

**Figure 4:**
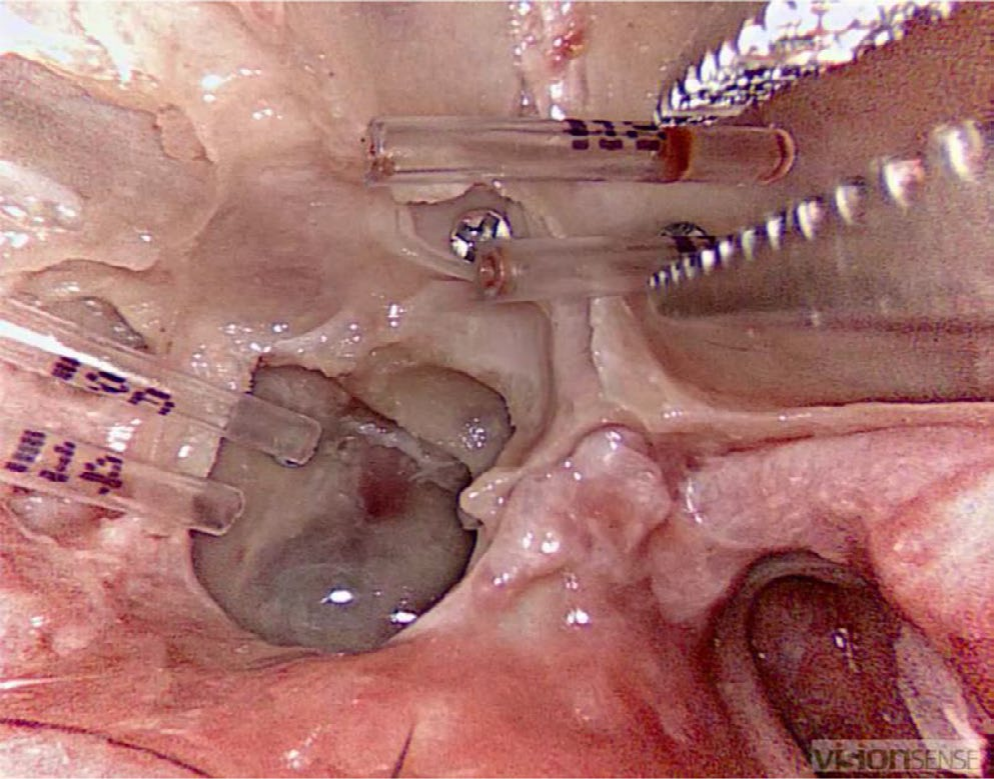
Fourth task: participant grasping one of the four translucent tubes

**Figure 5:**
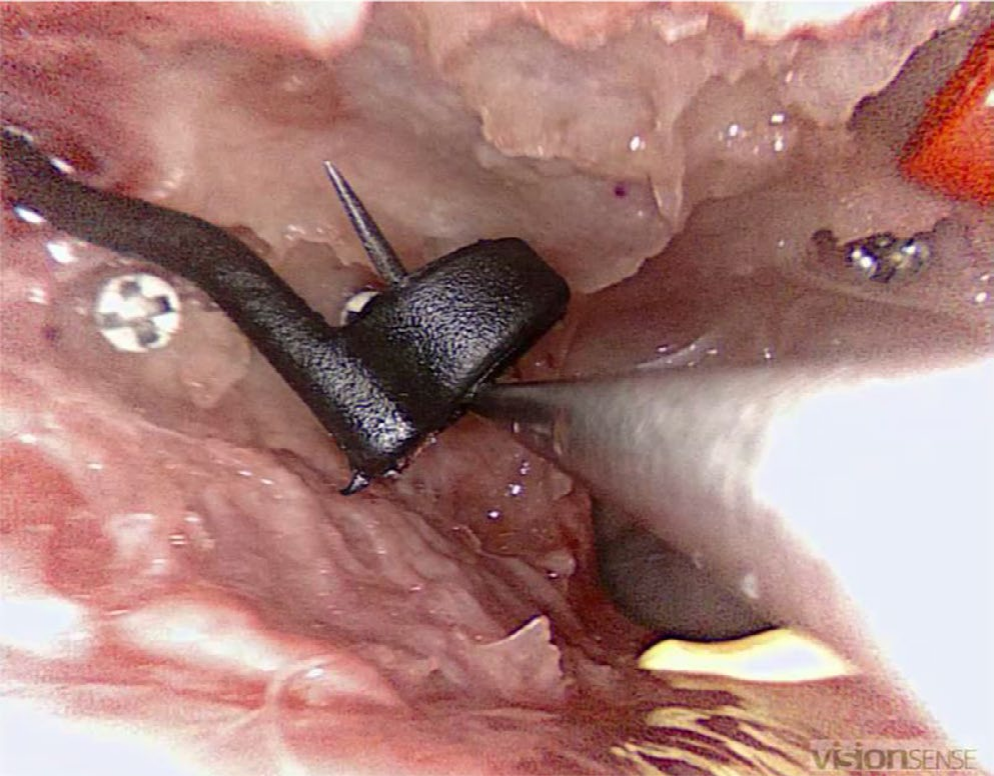
Fifth task: participant retrieving one of three ring‐shaped objects with the hooked instrument

The following objects, differing in visibility and spatial layout, were retrieved from the maxillary and sphenoid sinus using a straight, grasping forceps: coloured sponge discs, translucent tubes and ring‐shaped objects (EDGES™ Zig Aligna™, Fox International). A single, hooked, fine wound retractor was used to retrieve the Aligna's™ (Figures 3, 4 and 5).

### 2.6 Outcome measures

Primary outcome measures were as follows: surgical efficiency (defined as the time to task completion for all tasks), the total distance covered and average velocity towards the target (task 2), the total distance covered and average velocity inside the nasal cavity (task 3), and the surgical accuracy measured by total errors per task (all tasks). Errors were defined as identifying a wrong structure or making non‐functional contact with non‐target structures (task 1), pointing out or grasping an incorrect structure or object (tasks 2‐5), missing the correct object during an attempt to grasp or hook it or losing an object after grasping or hooking it (tasks 3‐5). To assess time scores and error rates, two researchers (EtD and HMH) individually reviewed video images of all tasks. A secondary, joint review was deemed necessary whenever the scores assigned differed by more than three errors per subtask. The average of the two scores was used as the final error score. Observers were unaware of participants' identity. The differing video output of the endoscopes, however, impeded blinding for endoscope type. Secondary outcome measures were as follows: endoscope characteristics and perceived task difficulty per endoscope type. These were assessed following each round of tasks using questionnaires. The following endoscope characteristics were assessed using a ten‐point Likert scale from 1 = “completely insufficient” to 10 = “perfect”: image sharpness, depth representation, colour representation, field of view size, discriminatory quality, suitability for accurate maneuvres and manoeuvrability. Perceived task difficulty per endoscope was measured using a five‐point Likert scale from 1 = “not difficult” to 5 = “very difficult.”

### 2.7 Statistical analysis

Demographic data of all participants were collected, including any experience considered relevant for endoscopic surgery (>30 endoscopic endonasal surgical procedures and/or rigid nasal endoscopies, carried out autonomously for more than half the procedures,^29^ or experience with action video games (playing more than 7 h/wk on average during their most active period of gaming)).^30^ To allow paired—intra‐user—analyses of performance scores, the results of both groups were combined for each endoscope type, thereby minimising the effect of the order in which the endoscopes were used. Analysis was performed using the Wilcoxon signed‐ranks test for paired results, after testing for homogeneity of distribution and equality of variances. After logarithmic conversion, the assumptions were met for all time and error scores, except the error scores of sponge removal. Results per task round were compared using a Mann‐Whitney *U* test for unpaired data. Because of the small sample size of this study, and the potential of type 2 errors, only descriptive statistics were used for comparison of results per endoscope type per task round. Statistical significance was assumed with a one‐tailed *P* value < .05. All statistical analysis was conducted using IBM SPSS Statistics version 22.0 (SPSS IBM, Inc).

## 3 RESULTS

### 3.1 Subjects

In total, 31 subjects were included: 12 (39%) residents (otorhinolaryngology [10], neurosurgery [1], general surgery [1]) and 19 (61%) medical students. One student withdrew from the study before starting. All subjects passed the Titmus circle test. Demographics of the 30 remaining participants are summarised in Table 1. Six of the 30 (20%) participants were considered as having relevant endoscopic experience. Five (17%) participants had relevant experience playing action video games. The resident in general surgery had considerable experience in laparoscopic surgery. Randomisation resulted in 17 (57%) participants in the group 2D first‐3D second and 13 (43%) participants in the group 3D first‐2D second. The groups did not differ significantly in age, function, handedness or relevant experience.

**Table 1:** Demographics

	2D‐3D (n = 17)	3D‐2D (n = 13)
Gender (M:F)	7:10	7:6
Dominant hand (R:L)	17:0	12:1
Age, median (range) in years	25 (20‐33)	26 (24‐34)
Medical student	12	7
Resident	5	6
Relevant experience (E:V)	4:4	2:1

### 3.2 Primary outcomes

#### 3.2.1 Efficiency

No significant differences between use of 2D HD and 3D HD endoscopy were found in time needed for completing the tasks “landmark identification” (*P* = .13), “pointing accuracy” (*P* = .32), grasping and retrieving “coloured sponge discs” (*P* = .26), “translucent tubes” (*P* = .39) or “ring‐shaped objects” (*P* = .30; Table 2). The total distances covered during the “target approach” (*P* = .38) and “grasping and retrieving coloured sponge discs” (*P* = .26) task were not significantly different. The average velocity towards target in “approaching targets” (*P* = .03) was significantly faster using 3D HD endoscopy, whereas the average velocity on the whole intranasal trajectory in “grasping and retrieving coloured sponge discs” (*P* = .20) did not differ significantly between the endoscopes (Table 3).

**Table 2:** Time and error scores per endoscope type (combined results of first and second round) shown as absolute score and standard deviation

Task	2D		3D		2D vs 3D	
	Time (SD)	Errors (SD)	Time (SD)	Errors (SD)	*P* † (Time)	*P* † (Errors)
1 Identification anatomical landmarks	11.8 (5.4)	1.9 (2.6)	12.8 (6.8)	2.0 (1.7)	.13	.19
2 Approaching targets	12.4 (5.0)	**0.7 (0.8)** *	13.4 (9.2)	**1.6 (1.4)** *	.32	**.00** *
3 Grasping and retrieving sponge discs	17.9 (7.2)	4.0 (3.2)	17.1 (7.9)	2.6 (2.5)	.26	.08
4 Grasping and retrieving translucent tubes	18.3 (5.7)	1.0 (1.3)	18.8 (8.4)	1.0 (1.0)	.39	.44
5 Grasping and retrieving ring‐shaped objects	29.6 (10.2)	3.0 (1.7)	29.0 (15.5)	2.4 (2.0)	.30	.08

* *P* < .05.

† One‐sided, exact significance.

**Table 3:** Average distance covered and average velocity towards the target (task 2) and during the whole trajectory inside the nose (task 3). Scores expressed as mean ± standard deviation (SD)

Group	2D		3D		2D vs 3D	
Task ↓	Distance (SD)	Velocity (SD)	Distance (SD)	Velocity (SD)	*P* † (Distance)	*P* * (Velocity)
1 Approaching targets	108.4 (33.6)	**9.2 (2.2)** *	103.5 (36.6)	**9.8 (2.5)** *	.38	**.03** *
2 Grasping and retrieving sponge discs	183.5 (53.1)	12.0 (4.3)	167.5 (37.7)	12.2 (4.0)	.12	.20

* *P* < .05.

† One‐sided, exact significance.

During the second task round, all tasks, except for “retrieving translucent tubes,” were completed significantly faster than during the first task round (*P* = .00‐.02). Users of 3D HD endoscopy completed all tasks the fastest, when 3D HD endoscopy was used during the second task round (2D first‐3D second). In contrast, users of 3D HD endoscopy needed the most time to complete each of the tasks, when 3D HD endoscopy was used in the first task round (3D first‐2D second).

The distance covered and mean velocity in “pointing accuracy” (*P* = .01 and .04) and distance covered in “sponge retrieving” (*P* = .02) improved significantly between the consecutive rounds. Again, participants using 3D HD endoscopy in the second round covered less distance and reached a higher average velocity than the other subgroups, although not reaching significance.

#### 3.2.2 Accuracy

With the exception of the “pointing accuracy” task, in which significantly more errors were made using 3D HD endoscopy (*P* = .00), error rates did not differ significantly (Table 3). Also, moderately strong positive correlations were found between time and error scores for “landmark identification” (*r* = .42; *P* < .01), “coloured sponge disc retrieving” (*r* = .37; *P* < .01) and “ring‐shaped object retrieving” (*r* = .40, *P* < .01).

### 3.3 Secondary outcomes

#### 3.3.1 Endoscope quality

Evaluation of endoscope characteristics showed significantly better scores for the 3D HD endoscope on image sharpness (*P* < .01), colour quality (*P* < .01) and depth representation (*P* = .02; Table 4). In addition, discriminatory power (*P* < .01) and the suitability to perform precise procedures (*P* < .01) were rated significantly better for the 3D HD endoscope, while no significant differences were found in the subjective field of view (*P* = .70) and manoeuvrability (*P* = .36).

**Table 4:** Subjectively rated endoscope characteristics per endoscope type, rated on a scale from 1‐10, with 10 being the best rating. Scores are expressed as mean ± standard deviation

Group	2D	3D	2D vs 3D
Task ↓	Mean rating	Mean rating	*P* † (rating)
Image sharpness	7.2 (0.9)	8.0 (1.0)	**.00** *
Depth representation	5.8 (1.7)	8.3 (1.2)	**.00** *
Colour representation	6.9 (1.1)	7.8 (1.1)	**.00** *
Size of sharp field of view	7.4 (0.9)	7.5 (0.7)	.35
Discriminatory power	7.2 (1.1)	8.2 (0.7)	**.00** *
Suitability for accurate procedures	7.0 (1.1)	8.2 (0.8)	**.00** *
Manoeuvrability	7.3 (1.1)	7.5 (0.8)	.33

* *P* < .05.

† One‐sided, exact significance.

#### 3.3.2 Task difficulty

For all five tasks, task difficulty was rated mild to moderate, with no significant differences between endoscope types. A trend favoured 3D HD endoscopy, with the most notable difference in difficulty rating in the retrieval of transparent tubes and ring‐shaped objects (Table 5).

**Table 5:** Subjectively rated task difficulty, rated on a scale from 1‐5, with 5 being the highest difficulty. Scores expressed as mean ± standard deviation (SD)

Group	2D	3D	2D vs 3D
Task ↓	Difficulty (SD)	Difficulty (SD)	*P* † (Rating)
1 Identification anatomical landmarks	2.3 (1.1)	2.3 (1.0)	.50
2 Approaching targets	2.2 (0.9)	1.9 (0.9)	**.04** *
3 Grasping and retrieving sponge discs	2.4 (0.9)	2.3 (0.9)	.32
4 Grasping and retrieving translucent tubes	2.4 (0.8)	2.1 (0.8)	.06
5 Grasping and retrieving ring‐shaped objects	3.1 (0.8)	2.7 (0.8)	**.02** *

* *P* < .05.

† One‐sided, exact significance.

The data that support the findings of this study are available from the author (HMH) upon reasonable request.

## 4 DISCUSSION

### 4.1 Key findings

The present study tested the hypothesis that the use of 3D HD endoscopy improves surgical performance in endoscopic endonasal sinus surgery. The objective results show no significant differences in surgical efficiency and accuracy between 2D HD and 3D HD endoscopy. Subjectively, the imaging quality of the 3D HD endoscope was rated significantly better.

In endoscopically naïve participants, the use of 3D HD endoscopy neither significantly nor consistently increased endoscopic surgery efficiency. Only during the target approach task, the average velocity towards a target—one of the outcome measures of efficiency—was higher using 3D HD endoscopy. This was not interpreted as an increased surgical efficiency, since the higher velocity was associated with a significant increase in error rate.

### 4.2 Strengths of the study

The present study was the first study comparing 2D HD and 3D HD endoscopy specifically in ESS. Objective outcome measures were used, and a realistic surgical environment was offered by using a Thiel‐embalmed human specimen as surgical field. In contrast to more simplified models, in which participants can rely on relatively few depth cues, the use of Thiel‐embalmed specimens increased the availability of haptic feedback. This is important because surgical performance depends on the availability and quality of sensory feedback.^18^, ^31^, ^32^, ^33^, ^34^, ^35^ Also this more complex surgical environment offered valuable additional depth cues such as tissue texture and shading of intranasal structures.^36^, ^37^, ^38^ Therefore, the relative influence of stereopsis is reduced. Moreover, the presence of real nasal mucosa in the surgical field further increased the validity of this study by including a known weakness of 3D HD endoscopy that is present in clinical use. Soiling of 3D endoscope optics by mucus or blood causes blurring of the endoscope images. This negatively affects the imaging quality of 3D endoscopes more that of 2D endoscopes, especially 3D endoscopes using insect eye technology.^7^, ^39^

### 4.3 Comparison with other studies

In recent past, various studies with a design similar to the present study assessed the influence of 3D endoscopy on EES performance.^11^, ^14^, ^17^, ^18^, ^22^, ^40^ A number of these studies report improved performance with the use of 3D endoscopy, in contrast with results of the present study. These differing results can be explained by differences in study setup. It should be noted that the results of our study should only be framed in the context of ESS, since key aspects of skull base surgery were not included in our study design. In general, prior studies assessed performance in simplified surgical environments, such as box trainers and dry anatomical models, and mostly used low complexity pointing tasks.^11^, ^14^, ^17^, ^18^, ^40^ In more complex surgical environments, endoscopic surgery tasks require active manoeuvring through a complex visual environment. The result is an increased availability of monocular depth cues, reducing the dependence on stereopsis.^36^, ^37^, ^38^ For example, Shah et al^14^ found that significantly more nerve hook placement tasks were successfully performed with 3D endoscopy in a box trainer model, using success or failure as outcomes. The authors found no differences in time to task completion or error rates during ring transfer and incision tasks. Fraser et al^40^ compared EES performance between 3D endoscopy and 2D endoscopy during endoscopic sellar floor removal and biopsy tasks, performed in a custom build test box. Cutting efficiency was significantly higher with 3D endoscopy during the second round of tasks, whereas time and error scores were comparable. In a third and more recent study by Rampinelli et al,^18^ participants performed a grasping and dissection movement task in a 3D printed skull model. No significant benefit of 3D endoscopy was found for inexperienced participants, which is confirmed by the present studies results. In the expert group, however, task completion time was significantly reduced.^18^ The results of these three studies indicate that both surgical environment, task complexity and endoscopic surgery experience influence performance and its relation to endoscopic imaging technique.

An additional explanation for the difference in results between prior studies and the present study is that the endoscope is used statically, rather than dynamically in most neurosurgical EES procedures.^14^ The ability to move the endoscope through the surgical field provided participants with dynamic depth cues, available in both 2D and 3D endoscopy.^6^, ^14^, ^41^

Up till now, only two previous studies used instrument tracking as objective outcome measure for endoscopic surgery performance. Inoue et al^12^ assessed instrument path length during endoscopic surgery tasks in a dry model for transsphenoidal surgery. In line with the present study, no significant differences were found between the use of 2D HD and 3D HD endoscopy during two pointing tasks. More recently, Rampinelli et al^18^ used instrument tracking to assess trajectories in grasping and dissection movement tasks, performed on a dry anatomical model. Two out of three of their indices of movement efficiency, the third derivative of the trajectory curve function and deviation from the average ideal curve, did not differ significantly between 3D standard definition (SD) and 2D HD endoscopy. The third index, representing the deviation from the ideal trajectory, showed that movement accuracy significantly improved for 3D endoscopy by expert users, whereas it significantly worsened in non‐experts. The authors suggest that experts translate the additional visual information into more effective instrument movement, whereas the non‐expert participants feel more confident, but lack the ability to translate the additional information into improved instrument use. A positive relation between visual information processing and expertise is also shown by Jarodzka et al^42^ supporting the relation suggested by Rampinelli et al^18^ The results of the present study show a significantly higher average velocity towards the targets, in combination with a significant increase in error rate when 3D HD endoscopy was used during the instrument placement task. This could well be the result of relative inability of inexperienced users to process the more complex visual information of the 3D HD endoscope. It could also indicate that 3D HD endoscopy provides a false sense of confidence to inexperienced users, although the latter was not supported by results of our study. Only positive correlations were found between time to task completion and error rates.

The subjective superiority of 3D endoscopy in EES has been widely reported by both clinical and laboratory studies and is confirmed by the subjective results of the present study.^5^, ^9^, ^10^, ^11^, ^12^, ^17^, ^28^, ^40^ The present study showed significantly better rating of depth perception, discriminatory power and the value in performing delicate procedures using the 3D HD endoscope. These results specifically confirm studies of 3D endoscopy in ESS by Manes et al^5^ and Albrecht et al.^9^ The former found enhanced depth perception and better orientation with 3D SD endoscopy compared with 2D HD endoscopy, whereas the latter found improved depth perception and comfort of use with 3D HD endoscopy and enhanced image sharpness compared with that of 2D HD.^5^

### 4.4 Drawbacks

The use of inexperienced participants can be regarded as a limitation of the present study, since it limits the transferability of the results to the clinical setting in which 3D HD endoscopy will be adopted by experienced endoscopic surgeons. Within a limited time window to perform the endoscopic tasks on the Thiel‐embalmed specimens, we chose to maximise the group sizes of two groups with inexperienced participants. This increased the reliability of our results, at the cost of adding comparison groups with endoscopically experienced participants. Nevertheless, the use of inexperienced participants was thought to exclude the effect of prior experience with 2D endoscopy. Experience in translating 2D images into a 3D impression of the surgical field, which is present in experienced surgeons, could unequally benefit 2D endoscopy, decreasing chances of finding a benefit of 3D endoscopy.^3^, ^22^, ^23^, ^43^ Also, the effect of 3D endoscopy on endoscopic surgery training could be analysed in inexperienced subject, although in the present study, the limited number of groups and relatively limited group size hindered thorough analysis of learning curves. Increased group sizes, the addition of control groups and comparison with experienced surgeons could provide further valuable information on 3D HD endoscopy. Also, future studies could possibly refine instrument tracking to objectively assess surgical performance. Target overshoot, air drilling and drilling depth, distance covered and number of movements all have previously been used.^3^, ^12^, ^18^, ^44^ The present study was the first to use average velocity to assess surgical efficiency. Optimal trajectories and measures of movement efficiency that are suitable for ESS should be developed and tested. Finally, as the evolution of endoscopic imaging techniques continues, future research should be used to identify the optimal application of new technologies, both 2D and 3D.

### 4.5 Clinical applicability

The present results indicate that in a realistic surgical environment, when performing complex surgical tasks, surgical performance is comparable with 2D HD and 3D HD endoscopy. The choice for either endoscope type should depend on the specific demands of a surgical procedure, and preference of the surgeon. Decisions on which endoscopy type to use can also be made based on the general difference in field of view between 2D and 3D endoscopes. The field of view of the 3D HD endoscope used in the present study was 31% smaller in the horizontal and 18% in the vertical direction than that of the 2D HD endoscope. The larger field of view of the 2D endoscopes could be preferable during procedures that require overview, whereas the addition of stereopsis can be preferred in procedures where the endoscope is used statically and overview is of lesser importance.^7^, ^24^

In inexperienced surgeons, training in ESS should be focused both on overcoming the difficulties of manoeuvring the endoscope through the complex surgical field, and on using the abundant visual and haptic feedback, regardless of the endoscope type used.

## 5 CONCLUSION

The use of 3D HD endoscopy in ESS did not significantly improve surgical performance of inexperienced surgeons compared with the use of 2D HD endoscopy, despite subjective superiority of the 3D HD endoscope. When choosing a specific endoscopic imaging technique, it is recommended to take the specific circumstances in which EES will be performed into account. Surgical field characteristics and surgical techniques are likely to influence any additional value of 3D HD endoscopy. To further assess current and future endoscopic imaging techniques, surgical navigation can be used as a specific and objective performance measure.

## Data Availability

The data that support the findings of this study are available from the author (HM.H) upon reasonable request.

## References

[1] Castelnuovo P , Dallan I , Battaglia P , Bignami M . Endoscopic endonasal skull base surgery: past, present and future. Eur Arch Oto‐Rhino‐Laryngol. 2010;267(5):649‐663. 10.1007/s00405-009-1196-0 20063006

[2] Garzaro M , Zenga F , Raimondo L , et al. Three‐dimensional endoscopy in transnasal transsphenoidal approach to clival chordomas. Head Neck. 2016;38:E1814‐E1819. 26698603 10.1002/hed.24324

[3] Taffinder N , Smith SGT , Huber J , Russell RCG , Darzi A . The effect of a second‐generation 3D endoscope on the laparoscopic precision of novices and experienced surgeons. Surg Endosc. 1999;13(11):1087‐1092. 10556444 10.1007/s004649901179

[4] Crosthwaite G , Chung T , Dunkley P , Shimi S , Cuschieri A . Comparison of direct vision and electronic two‐ and three‐ dimensional display systems on surgical task efficiency in endoscopic surgery. Br J Surg. 1995;82(6):849‐851. 7627529 10.1002/bjs.1800820640

[5] Manes RP , Barnett S , Batra PS . Utility of novel 3‐dimensional stereoscopic vision system for endoscopic sinonasal and skull‐base surgery. Int Forum Allergy Rhinol. 2011;1(3):191‐197. 22287372 10.1002/alr.20012

[6] Pennacchietti V , Garzaro M , Grottoli S , et al. Three‐dimensional endoscopic endonasal approach and outcomes in sellar lesions: a single‐center experience of 104 cases. World Neurosurg. 2016;89:121‐125. 26836697 10.1016/j.wneu.2016.01.049

[7] Barkhoudarian G , Romero ADCB , Laws ER . Evaluation of the 3‐dimensional endoscope in transsphenoidal surgery. Oper Neurosurg. 2013;73(1):74‐76. 10.1227/NEU.0b013e31828ba962 23407288

[8] Brown SM , Tabaee A , Singh A , Schwartz TH , Anand VK . Three‐dimensional endoscopic sinus surgery: feasibility and technical aspects. Otolaryngol Neck Surg. 2008;138(3):400‐402. 10.1016/j.otohns.2007.12.007 18312893

[9] Albrecht T , Baumann I , Plinkert PK , Simon C , Sertel S . Three‐dimensional endoscopic visualization in functional endoscopic sinus surgery. Eur Arch Oto‐Rhino‐Laryngol. 2016;273(11):3753‐3758. 10.1007/s00405-016-4040-3 27094054

[10] Catapano G , de Notaris M , Di Maria D , et al. The use of a three‐dimensional endoscope for different skull base tumors: results of a preliminary extended endonasal surgical series. Acta Neurochir (Wien). 2016;158(8):1605‐1616. 27278644 10.1007/s00701-016-2847-8

[11] Marcus HJ , Hughes‐Hallett A , Cundy TP , et al. Comparative effectiveness of 3‐dimensional vs 2‐dimensional and high‐definition vs standard‐definition neuroendoscopy: a preclinical randomized crossover study. Neurosurgery. 2014;74(4):375‐381. 24220007 10.1227/NEU.0000000000000249 PMC4053590

[12] Inoue D , Yoshimoto K , Uemura M , et al. Three‐dimensional high‐definition neuroendoscopic surgery: a controlled comparative laboratory study with two‐dimensional endoscopy and clinical application. J Neurol Surg Part A Cent Eur Neurosurg. 2013;74(06):357‐365. 10.1055/s-0033-1345100 23888482

[13] Roth J , Singh A , Nyquist G , et al. Three‐dimensional and 2‐dimensional endoscopic exposure of midline cranial base targets using expanded endonasal and transcranial approaches. Neurosurgery. 2009;65(6):1116‐1130. 19934971 10.1227/01.NEU.0000360340.85186.7A

[14] Shah RN , Leight WD , Patel MR , et al. A controlled laboratory and clinical evaluation of a three‐dimensional endoscope for endonasal sinus and skull base surgery. Am J Rhinol Allergy. 2011;25(3):141‐144. 21679524 10.2500/ajra.2011.25.3593

[15] Zaidi HA , Zehri A , Smith TR , Nakaji P , Laws ER . Efficacy of three‐dimensional endoscopy for ventral skull base pathology: a systematic review of the literature. World Neurosurg. 2016;86:419‐431. 26463398 10.1016/j.wneu.2015.10.004

[16] Sørensen SMD , Savran MM , Konge L , Bjerrum F . Three‐dimensional versus two‐dimensional vision in laparoscopy: a systematic review. Surg Endosc. 2016;30(1):11‐23. 25840896 10.1007/s00464-015-4189-7

[17] Kawanishi YU , Fujimoto Y , Kumagai N , et al. Evaluation of two‐ and three‐dimensional visualization for endoscopic endonasal surgery using a novel stereoendoscopic system in a novice: a comparison on a dry laboratory model. Acta Neurochir (Wien). 2013;155(9):1621‐1627. 23686635 10.1007/s00701-013-1757-2

[18] Rampinelli V , Doglietto F , Mattavelli D , et al. Two‐dimensional high definition versus three‐dimensional endoscopy in endonasal skull base surgery: a comparative preclinical study. World Neurosurg. 2017;105:223‐231. 28578115 10.1016/j.wneu.2017.05.130

[19] Cicione A , Autorino R , Breda A , et al. Three‐dimensional vs standard laparoscopy: comparative assessment using a validated program for laparoscopic urologic skills. Urology. 2013;82(6):1444‐1450. 24094658 10.1016/j.urology.2013.07.047

[20] Feng X , Morandi A , Boehne M , et al. 3‐Dimensional (3D) laparoscopy improves operating time in small spaces without impact on hemodynamics and psychomental stress parameters of the surgeon. Surg Endosc. 2015;29(5):1231‐1239. 25673344 10.1007/s00464-015-4083-3

[21] Storz P , Buess GF , Kunert W , Kirschniak A . 3D HD versus 2D HD: Surgical task efficiency in standardised phantom tasks. Surg Endosc. 2012;26(5):1454‐1460. 22179446 10.1007/s00464-011-2055-9

[22] Tabaee A , Anand VK , Fraser JF , et al. Three‐dimensional endoscopic pituitary surgery. Neurosurgery. 2009;64(suppl_5):ons288‐ons295. 10.1227/01.NEU.0000338069.51023.3C 19404107

[23] Votanopoulos K , Brunicardi FC , Thornby J , Bellows CF . Impact of three‐dimensional vision in laparoscopic training. World J Surg. 2008;32(1):110‐118. 17992561 10.1007/s00268-007-9253-6

[24] Van Gompel JJ , Tabor MH , Youssef AS , et al. Field of view comparison between two‐dimensional and three‐dimensional endoscopy. Laryngoscope. 2014;124(2):387‐390. 23712924 10.1002/lary.24222

[25] Thiel W . The preservation of the whole corpse with natural color. Ann Anat. 1992;174(3):185‐195. 1503236

[26] Eisma R , Lamb C , Soames RW . From formalin to thiel embalming: What changes? One anatomy department's experiences. Clin Anat. 2013;26(5):564‐571. 23408386 10.1002/ca.22222

[27] Turri‐Zanoni M , Battaglia P , Karligkiotis A , et al. Transnasal endoscopic partial maxillectomy: OPERATIVE nuances and proposal for a comprehensive classification system based on 1378 cases. Head Neck. 2017;39(4):754‐766. 28032687 10.1002/hed.24676

[28] Raheja A , Kalra R , Couldwell WT . Three‐dimensional versus two‐dimensional neuroendoscopy: A preclinical laboratory study. World Neurosurg. 2016;92:378‐385. 27216923 10.1016/j.wneu.2016.05.031

[29] Laeeq K , Lin SY , Varela DADV , Lane AP , Reh D , Bhatti NI . Achievement of competency in endoscopic sinus surgery of otolaryngology residents. Laryngoscope. 2013;123:2932‐2934. 24122507 10.1002/lary.23509

[30] Schlickum MK , Hedman L , Enochsson L , Kjellin A , Felländer‐Tsai LI . Systematic video game training in surgical novices improves performance in virtual reality endoscopic surgical simulators: a prospective randomized study. World J Surg. 2009;33(11):2360‐2367. 19649553 10.1007/s00268-009-0151-y

[31] He B , Giedraitis V , Ligers A , et al. Learning to use minimal access surgical instruments and 2‐dimensional remote visual feedback: How difficult is the task for novices? Adv Heal Sci Educ. 2002;7(2):117‐131. 10.1023/a:1015700526954 12075144

[32] Nisky I , Huang F , Milstein A , et al. Perception of stiffness in laparoscopy—the fulcrum effect. Stud Health Technol Inform. 2012;173:313‐319. 22357009 PMC4102265

[33] Gnanaseelan R , Gonzalez DA , Niechwiej‐Szwedo E . Binocular advantage for prehension movements performed in visually enriched environments requiring visual search. Front Hum Neurosci. 2014;8:959. 25506323 10.3389/fnhum.2014.00959 PMC4246685

[34] Bakker NH , Fokkens WJ , Grimbergen CA . Investigation of training needs for functional endoscopic sinus surgery (FESS). Rhinology. 2005;43(2):104‐108. 16008064

[35] Elliott D , Hansen S . Visual regulation of manual aiming: a comparison of methods. Behav Res Methods. 2010;42(4):1087‐1095. 21139176 10.3758/BRM.42.4.1087

[36] Frisby J , Shah J , Buckley D , Darzi A . Depth cue reliance in surgeons and medical students. Surg Endosc Other Interv Tech. 2003;17(9):1472‐1474. 10.1007/s00464-002-9178-y 12802650

[37] Glickfeld LL , Olsen SR . Higher‐order areas of the mouse visual cortex changes may still occur. Ann Rev Vis Sci. 2017;3(1):251‐273. 28746815 10.1146/annurev-vision-102016-061331

[38] Hanna GB , Cresswell AB , Cuschieri A . Shadow depth cues and endoscopic task performance. Arch Surg. 2002;137(10):1166‐1169. 12361428 10.1001/archsurg.137.10.1166

[39] Seong SY , Park SC , Chung HJ , Cho H‐J , Yoon J‐H , Kim C‐H . Clinical comparison of 3D endoscopic sinonasal surgery between ‘Insect Eye’ 3D and ‘Twin Lens’ 3D endoscopes. J Rhinol. 2016;23(2):102‐109.

[40] Fraser J , Allen B , Anand V , Schwartz T . Three‐dimensional neurostereoendoscopy: subjective and objective comparison to 2D. Minim Invasive Neurosurg. 2009;52(1):25‐31. 19247901 10.1055/s-0028-1104567

[41] Bolzoni Villaret A , Battaglia P , Tschabitscher M , et al. A 3‐dimensional transnasal endoscopic journey through the paranasal sinuses and adjacent skull base. Oper Neurosurg. 2014;10(1):116‐120. 10.1227/NEU.0000000000000172 24064484

[42] Jarodzka H , Scheiter K , Gerjets P , van Gog T . In the eyes of the beholder: How experts and novices interpret dynamic stimuli. Learn Instr. 2010;20(2):146‐154.

[43] Blavier A , Gaudissart Q , Cadière G‐B , Nyssen A‐S . Comparison of learning curves and skill transfer between classical and robotic laparoscopy according to the viewing conditions: implications for training. Am J Surg. 2007;194(1):115‐121. 17560922 10.1016/j.amjsurg.2006.10.014

[44] Al‐Saud LM , Mushtaq F , Mirghani I , et al. Drilling into the functional significance of stereopsis: the impact of stereoscopic information on surgical performance. Ophthalmic Physiol Opt. 2017;37(4):498‐506. 28656672 10.1111/opo.12393 PMC5519940

